# Stakeholder Consultation to Establish Research Priorities for Specialist Dementia Nursing in the United Kingdom

**DOI:** 10.1111/hex.14165

**Published:** 2024-10-01

**Authors:** Emma Wolverson, Amy Pepper, Karen Harrison Dening

**Affiliations:** ^1^ Dementia UK, One Aldgate London UK

**Keywords:** Admiral Nursing, dementia, Nominal Group Technique, research priorities, specialist nursing

## Abstract

**Introduction:**

In the United Kingdom, there are a growing number of specialist dementia nurses called Admiral Nurses. Admiral Nurses, supported in their professional development and clinical supervision by the charity Dementia UK, work with families affected by dementia using a relationship‐centred approach. Given the growing need for this type of support, Dementia UK is committed to research that will expand the evidence base for Admiral Nursing. This article describes a stakeholder consultation to identify research priorities for Admiral Nursing for the next 3 years (2023–2026).

**Methods:**

We adopted a participatory approach using an adapted Nominal Group Technique and priority‐setting workshop. All elements of the process were designed in consultation with a steering group comprising a range of stakeholders, including people with dementia, carers, Admiral Nurses, Dementia UK staff and researchers. Stakeholders were identified as those who were likely to be affected by or interested in the emerging research priorities. Nominal groups were held both face‐to‐face and online. A total of 144 people shared their research priorities. Data generated through each nominal group were thematically analysed and then ranked in order of priority.

**Results:**

Four themes reflecting research priority areas were taken to a priority‐setting workshop for consideration. This resulted in three research priorities for Admiral Nursing: (1) people with dementia who live alone and carers who provide support from a distance; (2) people living with young onset and rarer dementia and their families; and (3) people living with multiple health conditions alongside dementia, including mental health problems. Risk, diversity and the effectiveness of Admiral Nursing were strands that ran throughout these themes.

**Conclusions:**

We identified shared research priorities for Admiral Nursing using a rigorous, consensus‐driven approach involving key stakeholders. These priorities reflect a desire to ensure that Admiral Nursing services reach the most vulnerable people living with dementia and their families and respond to the widening health and social care inequalities faced by this group.

**Patient and Public Contribution:**

People with dementia and carers were involved in the design of this process as members of our steering group and through consultation on our initial plans with Dementia UK's Lived Experience Advisory Panel (LEAP). People living with dementia and carers also participated in consultation groups to share their views on research priorities. All stakeholders were invited to share feedback on the themes as part of the analysis and interpretation of the priorities, and a meeting was held with LEAP to discuss the emerging priorities.

## 1 Introduction

The definition of specialist and advanced nursing roles varies [1], but there is broad consensus that these roles involve nurses who have received education at degree level or above and possess advanced knowledge, skills, competencies and experience to focus care on a particular disease group or population.

Specialist nurses in the United Kingdom play a crucial role in managing complex conditions across various health care settings, including primary, secondary, community, acute and social care [2]. Dementia serves as an illustrative example of such a complex condition; although research is scant, there is evidence that dementia specialist nurses improve outcomes for both people with dementia and their family carers [3, 4].

Approximately 944,000 people are living with dementia in the United Kingdom as of 2023 [5], supported by over 700,000 unpaid carers. Further projections suggest that this number could increase to 1.7 million by the year 2050 [6], emphasising the growing demand for support for both those with dementia and their families. However, across the United Kingdom, there is variation in the quality and type of support people living with dementia and their families receive [7], including access to specialist dementia nursing.

In the United Kingdom, there are a growing number of specialist dementia nurses called Admiral Nurses. Admiral Nurses, supported by the charity Dementia UK in their continuing professional development and clinical supervision, work with the whole family affected by dementia utilising a case management approach [8]. They provide coordination of care and evidence‐based interventions to help families cope with the symptoms of dementia. They also provide consultancy, supporting other generalist professionals in the care of families affected by dementia through bespoke education, mentoring and supervision. Admiral Nurses are hosted by a range of organisations across various care settings (including acute hospitals, hospices, community, primary and social care) and offer a national helpline.

Admiral Nursing has become even more important after COVID‐19 as a direct result of widening health inequalities and service fragmentation has meant that people with dementia in the United Kingdom are increasingly struggling to access care and support when they need it [7]. Given this growing need and to ensure that every family affected by dementia can access this vital resource, Dementia UK is committed to research that will expand the evidence base for Admiral Nursing to support commissioning. Although descriptive and qualitative evidence suggests that Admiral Nurses are valued by family carers, the impact of their work has not yet been clearly established.

We aimed to identify the research priorities for Admiral Nursing for the next 3 years (2023–2026). This identification and prioritisation would inform decisions on where Dementia UK should focus its research activities and research collaborations in respect of Admiral Nursing.

## 2 Method

To ensure that the identified priorities had the greatest value for Admiral Nursing and were of the highest importance to people with dementia and their families, a stakeholder involvement approach was adopted. A robust and systematic approach to priority setting was followed to ensure that we involved stakeholders effectively, inclusively and transparently.

We adopted a participatory approach as the theoretical frame of reference [9] seeking to foster collaboration, empower participants and facilitate informed decision‐making. We used an adapted Nominal Group Technique (NGT) [10], a structured evaluative methodology. NGT was selected because it facilitates equal participation allowing all opinions to be considered and promoting inclusivity, provides a clear and systematic process to enhance collaboration and keep discussions focused and can be delivered with less time and resources than other consensus methods, an important consideration for a charity. Within the groups, we followed the adapted online methods outlined by Smith et al. [11]. A steering group prioritisation phase was added once all the nominal groups (NGs) had been completed to bring the views of diverse stakeholders together whilst promoting inclusivity, engagement and transparency. NGTs have been used successfully in health care settings for those with impaired language, understanding and capacity [12, 13].

An expert steering group oversaw the project and was composed of people with dementia and carers recruited from an established patient and public involvement (PPI) group (*n* = 3), Admiral Nurses (*n* = 3), including the chief Admiral Nurse and CEO), Dementia UK staff from across the organisation (practice development, communications, research and policy) (*n* = 4), two trustees from Dementia UK (*n* = 2) and an external professor of health care research (*n* = 1). We used purposive sampling, inviting individuals via email and contacting those we felt were likely to be most informative in generating research priorities for Admiral Nursing. We targeted individuals with in‐depth knowledge of Admiral Nursing and sought to include experts from different backgrounds to ensure a well‐rounded discussion.

### 2.1 Identifying NG Stakeholders

Stakeholders were identified as those who were likely to be affected by or interested in the emerging research priorities (Table 1).

**Table 1:** Sampling and identification of stakeholders for nominal groups.

Stakeholder group	Approach to identification and sampling
People with dementia and family carers and supporters	Identified via the Dementia UK Lived Experience Advisory Panel (LEAP), a panel that collaborates with and advises Dementia UK on many aspects of their work. All members of LEAP were invited and participated.
Admiral Nurses	All nurses (~450) were invited via email. Nurses contacted the research team if they wished to participate. Places were allocated on a first come, first‐served basis, taking care to ensure groups were representative of the different settings where Admiral Nurses work. Existing literature advocates 8–10 participants for an NG [11]. We over‐recruited to accommodate dropouts and aimed to recruit 30 nurses across three groups.
Dementia UK staff	Staff were purposely sampled from departments in business development, marketing, fundraising and policy. An invitation was emailed to each department inviting those interested to contact the research team and book onto a group. Participants were allocated on a first come, first‐served basis and aimed to recruit 30 staff from across departments.
Researchers in dementia care	Researchers in dementia care from UK universities with established links to Admiral Nursing were purposively sampled to ensure representation of different disciplines (nursing, social work, etc.), research across the dementia care trajectory and those known to work with diverse communities. Eight researchers were contacted and invited to take part.
Managers and commissioners of Admiral Nurse services	Host organisations that were managers or commissioners of Admiral Nursing services, such as NHS or social care, were purposively sampled across both health and social care as it was felt that research priorities might differ in these settings. Our intention was to recruit 8–10 people for one NG.

### 2.2 Conduct of the NGs

NGs were held face‐to‐face or virtually using an online platform (by E.W., K.H.D. and A.P.). All NGs were conducted according to a predetermined schedule, which included a group presentation introducing the aims of the consultation and ground rules for participation. No preparatory work was required, and groups lasted no longer than 1 h.

#### 2.2.1 Stage 1: Silent Generation of Ideas (Research Priorities; 5 min)

Stakeholders were asked to silently and independently generate an unlimited number of ideas in response to the question ‘What are the research priorities for Admiral Nursing now and in the future?’ These were then shared in ‘post‐it’ notes in the face‐to‐face meetings or, for the online group, typed into a chat box and sent to one of the researchers (A.P.). To maintain engagement, especially for people with dementia, researchers offered periodic reminders of the group's purpose and the task at hand throughout the session.

#### 2.2.2 Stage 2: Discussion, Clarification and Generation of Themes (40 min)

This stage involved a structured and time‐limited discussion and clarification of all ideas generated. During the discussion, suggestions that appeared similar were merged and labelled as new items (taking care not to lose original information or detail), for example, cost savings and core outcome measures merged into an agreed theme about measuring impact. This process of merging and labelling ideas was undertaken with iterative checking to ensure that individuals did not feel their ideas had been lost. Facilitators used open‐ended questioning (e.g., ‘Why is this a priority for Admiral Nursing?’ or ‘What is it specifically that Admiral Nurses need to know?’) to encourage a focus on research priorities that could be addressed by Admiral Nurses and not related to dementia care research more generally.

#### 2.2.3 Stage 3: Ranking and Prioritising Themes (15 min)

Finally, stakeholders voted independently on the themes and prioritised them with a first, second or third rank, with first being the highest priority and third the lowest. For face‐to‐face groups, a ‘voting slip’ was individually completed and collated at the end of the NG. For online groups, Mentimeter, an online polling software, was used for ranking priorities.

The approach was piloted at a Dementia UK staff ‘away day’. The pre‐NG presentation outlining the purpose of the NG was delivered to the entire group (see findings for the numbers of stakeholders) and then stakeholders were divided into smaller groups (8–10 members) to facilitate the NG process. All other NG groups consisted of 8–10 people, except for groups for people with dementia and carers, which had between 3 and 6 people, following guidelines on managing friendly focus groups for people affected by dementia [14].

### 2.3 Data Analysis

Two forms of data were generated through stages 1 and 2 of the NG process:

A. Aggregated themes from all groups.
B. Aggregated ranking from some groups as some stakeholders with dementia struggled with the ranking process.

The individually generated research priorities, research themes and questions from group discussions (data A) were thematically analysed [15] to facilitate cross‐group comparison. Analysis was conducted by two researchers (E.W. and K.H.D.) who each coded these data independently and then came together to discuss and agree on corresponding themes. Through discussion with a third researcher (A.P.), the themes were refined and named.

The ranked scores (data B) were initially gathered and consolidated based on stakeholder group prioritisation. As themes were identified, they were checked against priority ratings to ensure that they reflected the highest priority areas. The researchers had experience with qualitative methods and NGs. The trustworthiness of the analysis was ensured through iterative engagement with the data.

### 2.4 Priority‐Setting Workshop

The steering group came together for a full‐day workshop to identify final research priorities. The group was provided with the themes, rankings and considerations to guide the research priorities (see later) to read ahead of the meeting. Researchers introduced the day with an outline of purpose and a summary of all findings, providing opportunities for clarification and questions. Field notes were recorded on flip charts to visually share and validate discussions. One of the research team (A.P.) ensured that people with dementia involved were able to share their views and offer feedback in a sensitive and timely manner.

#### 2.4.1 Considerations to Guide the Research Priorities

To focus effort and resources within the 3‐year time frame of the strategy, priorities were mapped against the core aims and purpose of Admiral Nursing and Dementia UK, relevance to dementia policy and limited to priorities that could be addressed by research.

A document outlining the agreed themes was shared via email after workshop meeting, seeking agreement that the priorities and their wording were ready to share and used during subsequent consultation.

### 2.5 Consultation of Themes Developed Through NGs

The document outlining the agreed themes was then shared with all stakeholders. The purpose of member consultation was to obtain feedback from everyone involved in the NG process. No large changes were suggested at this point, but changes were made to the wording and emphasis of the document, which resulted in a final version.

### 2.6 Ethical Considerations

This approach of stakeholder activity is patient and public engagement rather than primary research [16, 17]. The project did not meet the Health Research Authority (HRA) definition of research because stakeholders were not randomised to different groups, we did not change treatment or care and our intention was not to generalise our findings beyond Admiral Nursing. Therefore, ethical approval was not required; however, we abided by the ethical principles of the Social Research Association [18] throughout.

## 3 Results

In total, 144 people shared their views during the priority‐setting work between January 2023 and August 2023 (Table 2). A total of 11 consultation groups were held with 74 people overall and an additional 39 Admiral Nurses and 31 Dementia UK staff took part during the away day.

**Table 2:** Number of stakeholders from each group.

Stakeholder group	Number of people
People with dementia and family carers and supporters from the Lived Experience Advisory Panel (LEAP), a panel that collaborates and advises Dementia UK on many aspects of their work.	32
Admiral Nurses	56
Dementia UK staff from departments including business development, marketing, fundraising and policy.	44
Researchers in dementia care from UK universities. Researchers were purposively sampled to ensure representation of different disciplines (nursing, social work, etc.), research across the dementia care trajectory and those known to work with diverse communities.	5
Host organisations that were managers or commissioners of Admiral Nursing services, such as NHS or social care.	5

### 3.1 Qualitative Data Analysis

Four themes were generated during the analysis. The first theme, ‘the impact and outcomes of Admiral Nursing’, reflected a desire for research that demonstrates the value of Admiral Nursing to continue to expand the profession. The second theme, ‘ensuring nobody faces dementia alone’, focused on vulnerable populations where it was felt that Admiral Nurses could make a real difference, but where more research was needed to extend the reach of the profession. In contrast, the themes ‘supporting people with young onset dementia at different stages of living with dementia’ and ‘supporting the complex and multifaceted needs of people with dementia and their families’ were priorities in areas where the specialist role of Admiral Nurses was well established and research was sought to demonstrate the value of the role alongside research to provide more evidenced base interventions for nurses.

Some stakeholders with dementia struggled with the ranking process, specifically holding all the themes in mind and allocating them consecutive numbers. Therefore, overall ranks are not presented; instead, we have provided commentary on the ranking including divergence and consensus across groups within the description of the themes.

### 3.2 The Impact and Outcomes of Admiral Nursing

This was a strong theme ranked first by Admiral Nurses, Dementia UK staff, health care professionals and researchers. However, only one person with dementia mentioned a desire for research that demonstrates the benefits and the impact of Admiral Nursing.

Discussions centred around the economic pressures on health services and the need to provide strong evidence of ‘value’ and in particular cost savings that Admiral Nursing can make to the health and social care system. Proposed questions included:

How much Admiral Nursing costs and how much it saves the NHS and Social Care?

(Admiral Nurse)

What are the economic benefits of Admiral Nursing?

(Dementia UK Staff)

What is the return on investment for Admiral Nurse Services?

(Person with Dementia)

Measuring the wider impact (beyond cost savings) that Admiral Nurses make on people with dementia and their families was also discussed, with a particular focus on demonstrating improvements in quality of life and in preventing hospital admissions.

We need more impact information and for this to be easily broken down e.g. regionally and by county.

(Dementia UK Staff)

What outcomes are important to Admiral Nurses and how do they measure them? How do these outcomes align with the outcomes of importance for the families?

(Researcher)

Admiral Nurses employ a range of interventions to support people with dementia and their family carers, and an area of particular interest was research to generate evidence of the effectiveness of these interventions. Research questions focused on whether effectiveness differed across the different settings and whether there were any specific interventions only used by Admiral Nurses.

Which interventions are most effective and can be delivered in different settings?

(Admiral Nurse)

Are there specific interventions … that that Admiral nurses are best placed to deliver?

(Admiral Nurse)

### 3.3 Ensuring Nobody Faces Dementia Alone

A significant focus of discussions across groups centred on the growing health inequalities experienced by people living with dementia and their families. This priority was ranked second by Dementia UK staff, researchers and health care professionals and fourth by Admiral Nurses. There was a desire to ensure that support from Admiral Nurses reached the most vulnerable people living with dementia and their families.

The need for Admiral Nursing now is huge, how can Admiral Nurses be more available in local communities?

(Admiral Nurse)

How do Admiral Nurses work with people from underserved communities?

(Person with dementia)

There were calls for research to establish the current reach of Admiral Nursing services, with the inference that such knowledge is known internally to Dementia UK and less so to external stakeholders.

How does the location of Admiral Nursing services map with diverse communities across the UK?

(Carer)

Stakeholders recognised that Admiral Nurses needed to be creative in engaging with groups such as those from minoritised communities, and that this might require innovative ways of working and the development of new skills.

How confident and prepared do admiral nurses feel to provide care for people from diverse communities?

(Researcher)

There was an awareness and a concern about the growing population of people living alone with dementia who were identified as facing significant challenges in accessing Admiral Nurse services, as it was typically a carer or relative who made requests for support.

How do Admiral Nurses work with people with dementia who live alone?

(Person with dementia)

The pandemic had resulted in many changes to services for families affected by dementia, especially in their cessation during lockdown and then a failure to reinstate them. Stakeholders discussed how the health and social care landscape had changed because of COVID‐19 and questioned if we knew what people's needs were now.

How have family support needs changed? What do families need now compared to pre‐covid?

(Admiral Nurse)

Changes in family structures were also noted and required Admiral Nurses to work in new ways, particularly to engage and support carers who cared from a distance. This resulted in requests for evidence‐based interventions for Admiral Nurses working with distance carers.

### 3.4 Supporting People With Young Onset Dementia at Different Stages of Living With Dementia

Providing support to people living with young onset dementia and their families was recognised as an area that Admiral Nurses and staff at Dementia UK felt Admiral Nurses made a singificant difference. This priority was ranked third by Admiral Nurses and fourth by staff at Dementia UK. Although it was mentioned as important by some health care professionals and researchers in passing, it wasn't a theme that was put forward for final ranking. Supporting people with young onset dementia was an area where specialist nursing expertise was often recognised by other health care professionals. Working with people living with young onset dementia inevitably involved working with their family members and children and as such was seen to exemplify the Admiral Nursing model in working with complexity. Questions for research related to understanding the best ways to support families during transitions to residential care and palliative and end‐of‐life care.

How can Admiral Nurses support people with young onset dementia as they transition into residential care?

(Admiral Nurse)

Interventions to support younger carers and young children whose parents had dementia were also raised as a priority.

What are the best ways to support children when a parent has young onset dementia?

(Carer)

Staff at Dementia UK wanted research exploring ‘health economics in relation to young onset dementia’ to demonstrate the cost savings Admiral Nurses could make.

### 3.5 Supporting the Complex and Multifaceted Needs of People With Dementia and Their Families

This theme reflected discussions that Admiral Nurses, as specialists, aim to support people with multifaceted and complex needs. As a priority, it was rated second by Admiral Nurses and third by Dementia UK staff, but it was not mentioned by researchers, health care professionals, people with dementia or carers.

This included recognition that people with dementia often live with multiple health conditions, frailty and mental health conditions. The NG process highlighted how this group often experienced inequalities because of their dementia diagnosis, and this exacerbated other health conditions. The role of Admiral Nurses as advocates and case managers for this group was frequently mentioned. There was a desire for research to give evidence to the challenging health situations that some people with dementia and their families experience to highlight the specialist skill set of Admiral Nurses.

Can we evidence how Admiral Nurses support the frailty agenda?

(Dementia UK staff member)

How do we define complexity/levels of need in respect of Admiral Nursing caseloads and in different care settings?

(Dementia UK staff member)

There were calls to prioritise research that explored the role that Admiral Nurses play in preventing crisis situations from occurring, especially related to mental health crises, distressed behaviours and the role of Admiral Nurses in minimising the impact of co‐existing health conditions.

How do Admiral Nurses help people to avoid crises?

(Person with dementia)

What is the role of Admiral Nurses in preventing deterioration?

(Researcher)

Moreover, complexity is also related to the Admiral Nurse's role in working with whole families and a recognition that in response to societal shifts, family structures have become more intricate with more diverse family configurations and changing roles. Technological advances in communication tools and globalisation connect families across distances meaning that Admiral Nurses can be supporting carers all over the United Kingdom and the world.

Risk assessment and management was another significant layer of complexity in Admiral Nursing care, with nurses explaining that safeguarding concerns and risks had increased significantly during and since COVID‐19. People who live alone with dementia were seen as being particularly at risk and only coming to the attention of services in a crisis. The risk of carers who provide support from a distance and their ability to access support was an area that needed more consideration. Overall, there was a call to better understand how nurses assess and prioritise risks when faced with complex care situations.

I think a framework of urgency would be helpful.

(Admiral Nurse)

### 3.6 Final Priorities

The final steering group meeting set the three research priorities and developed an explanatory narrative, derived from consensus group data:

- People with dementia who live alone and families providing support from a distance.
  This priority reflects the desire to expand the reach of Admiral Nurses. People living alone with dementia are a growing population who face unique challenges and significant inequities in accessing support. The priority also embraces the call for research to develop evidence‐based interventions for Admiral Nurses who provide support to families providing care from a distance in response to changing family structures, societal norms and new technologies.
- People with young onset dementia and rarer dementias.
  This population was recognised as facing significant challenges in accessing care and support specific to their unique needs. Admiral Nurses work with whole families to support well‐being, and this priority reflects a desire to ensure that nurses have evidence‐based systemic interventions in their armoury. The term ‘rarer dementia’ was also embraced in this priority with an understanding that many people with young onset dementia will be diagnosed with a rarer form. Research must focus on how to provide continuity in care for this population and understanding key points of transition where Admiral Nursing interventions can make a difference.
- People living with multiple health conditions alongside dementia, including mental health problems.
  This priority reflects the specialism of Admiral Nurses and the often complex care needs of the people they support. People with multiple long‐term conditions and mental health problems often live in deprived areas and face challenges in accessing resources. Carers of those with multiple health conditions will have extra responsibilities and worries. Research is needed to demonstrate the role Admiral Nurses can play in preventing, minimising and managing chronic, long‐term conditions in their coordination of care.

It was agreed that all research conducted according to these priorities should also seek to demonstrate the benefits and cost savings that Admiral Nurses can make and focus, where possible, on intervention development. Diversity in terms of culture, race, ethnicity, sexuality and gender will be embraced across all research conducted in relation to these priorities with active and meaningful engagement of people from communities often excluded from research.

## 4 Discussion

The three priorities reflect a desire to ensure that the support of Admiral Nurses reaches those most vulnerable. In the context of widening health and social care inequalities, Admiral Nurses are working with increased complexity and risk, and these strands run throughout each priority. The necessity to demonstrate outcomes and cost savings in this climate of austerity is another common strand across three areas [19].

Clearly, these priorities are not isolated from each other; some of the most vulnerable people may well fit within all three of these priority areas. Within each priority, diversity and individual differences in all their forms must be embraced.

### 4.1 Living Alone and Families Caring From a Distance

The population of people in the United Kingdom who live alone is increasing, accounting for 30% of all households in 2022 [20]. People who live alone are 30% more likely to develop dementia compared to those who live with others [21].

In the United Kingdom, approximately 120,000 people are living alone with dementia [22]. There are many reasons why people with dementia live alone, and many people are happy with this arrangement [23]. However, research suggests that this population is at an increased risk of social isolation and loneliness and has significantly more unmet needs compared to those living with a carer [24]. These unmet needs often hasten a move to residential care [24].

There is little research on the best ways to support people who live alone. For Admiral Nurses, the desire for more evidence‐based interventions to support those who live alone may also reflect that historically, the model of Admiral Nursing was to provide support to the family carer and in doing so indirectly impact upon the outcomes of the person living with dementia. However, over time, the Admiral Nursing model moved to a relational model that supports the whole family system [25].

Not everyone who lives alone will have a carer, but many have families and friends providing support from a distance. Distance carers are a growing population who play a vital role in supporting people with dementia. Approximately 15%–20% of all informal caregivers, most of them adult children of ageing parents, provide care from a geographic distance [26]. Distance caring brings unique challenges, and research has demonstrated that whilst distance carers can experience personal growth and an enhanced relationship with the care recipient, they can also experience social isolation, distress and anxiety [27].

Our findings suggest that Admiral Nurses need evidence‐based interventions that take into consideration the distance care demands, challenges and burdens. Research suggests that distance carers need education on how to access available resources that will facilitate the ability of distance caring, coping strategies skills training and emotional support to deal with the challenges and struggles associated with distance caring, such as feelings of guilt, burden, anxiety and emotional distress [27].

### 4.2 People With Young Onset Dementia and Rarer Dementias

This population faces significant challenges in accessing care and support specific to their needs and yet has complex support needs requiring more intense care [28]. As such stakeholders in our consultation felt that the support of those with young onset and rarer forms of dementia was an area where specialist support should be expanded. Admiral Nurses reported that the support of people with young onset dementia and their families was an area where their status and expertise as specialist nurses were often recognised.

Given the high prevalence of mental health needs for people with young onset dementia [29], the need for skilled assessment of mental health needs and risk management was highlighted in this consultation as an area for research. This reflects the paucity of research on effective assessment and risk management tools in dementia care nursing.

The need to formulate holistic health care and support plans that recognise the needs of the whole family was also raised in the consultation, in particular, a need for evidence‐based interventions to maintain family bonds and support children was highlighted as research gaps. This is consistent with the conclusions from the Angela project on post‐diagnostic support needs [28].

### 4.3 People Living With Multiple Health Conditions Alongside Dementia, Including Mental Health Problems

This priority reflects the complex care needs of people living with dementia and the reality that most will also be living with multiple chronic health conditions alongside their dementia. As much of the medical profession and the NHS is organised around single diseases [19], Admiral Nursing offers the coordination of care to span a range of conditions and so, services and specialities. Specific research questions included a desire to evidence how Admiral Nurses can help people with dementia stay as independent as possible, enjoy a good quality of life and prevent, delay or minimise the disability of frailty. Our consultation also highlighted an awareness that carers of those with multiple health conditions, such as diabetes or frailty, often have extra responsibilities and worries and Admiral Nurses wanted more evidence‐based interventions to be able to provide appropriate support. The rising mental health needs of people in later life and the need for suitable assessments and interventions to prevent people from reaching mental health crisis was also deemed important.

### 4.4 Limitations

It is important to acknowledge the limitations of this work. Our sample allowed us to hear the voices of key stakeholders in shaping Admiral Nursing and we attempted to hear from people with dementia from diverse backgrounds; however, the sample is small and clearly not representative of the views of all family carers of people living with dementia. We were unable to recruit as many academics and commissioners as hoped. We did not follow up on the reasons for this and cannot report on why participation was lower among these groups, but it may have impacted how representative the priorities are of their views.

The process of ranking priorities was harder for people with dementia, despite attempts to make the ranking as simple and engaging as possible. Holding all the priorities in mind proved difficult for some. One participant at the end of a consensus meeting admitted rating in a random order for ease; we do not know how many other individuals with dementia may have done this. However, all individuals were able to contribute to the discussions and provide meaningful questions for research. We hope in publishing this work that we have demonstrated the value of engaging people with dementia in all aspects of research, including priority setting.

It could be questioned given that two priorities reflect areas where Admiral Nursing specialism is well recognised, whether these are research priorities or identified areas in care where the Admiral Nurse plays an important role. However, the desire to extend the research of Admiral Nursing based on population needs and changes (such as those living alone), for innovative solutions to changing family structures and to develop evidence‐based interventions are all valuable topics for research.

We acknowledge that the consultation was initiated, managed and funded by Dementia UK so may be construed as establishing a bias in the process of developing research priorities. However, we ensured that stakeholders also included external individuals, such as families who had not received Admiral Nursing services, host organisations, researchers in the field, service commissioners of services both with and without Admiral Nursing, to mitigate as much as possible from the groups being biased positively towards Admiral Nursing. It was more difficult for those not fully familiar with the role of the Admiral Nurse to discuss research priorities.

### 4.5 Next Steps

To generate high‐quality research in line with these three priorities, and as part of their research strategy (September 2023–September 2026), Dementia UK has committed to a number of actions, primarily to develop research collaborations to include people with dementia, their families, academic partners and Admiral Nurses in the three research priorities. Steering groups have been convened for each priority. Dementia UK will also invest in building the research capacity of Admiral Nurses, recognising the crucial role of Admiral Nurses in sharpening their profession.

## Author Contributions

**Emma Wolverson:** conceptualisation, investigation, funding acquisition, writing–original draft, methodology, formal analysis, project administration, visualisation, validation. **Amy Pepper:** conceptualisation, investigation, writing–review and editing, methodology, data curation, project administration, visualisation. **Karen Harrison Dening:** conceptualisation, investigation, writing–review and editing, methodology, visualisation, formal analysis, funding acquisition, validation.

## Ethics Statement

This approach to stakeholder activity is patient and public engagement rather than primary research [16, 17]. The project did not meet the Health Research Authority (HRA) definition of research because stakeholders were not randomised to different groups, we did not change treatment or care and our intention was not to generalise our findings beyond Admiral Nursing. Therefore, ethical approval was not required; however, we abided by the ethical principles of the Social Research Association [18] throughout.

## Conflicts of Interest

The consultation was initiated, managed and funded by Dementia UK, the charity that supports and develops Admiral Nurses.

## Data Availability

Data are available from the authors on request.
